# The Hospital World

**Published:** 1911-07-15

**Authors:** 


### THE HOSPITAL WORLD.

### The New Chaplain at Guy's.

At the recent general court at Guy's Hospital the Bishop of Southwark was appointed chaplain to the hospital.

### A Successful Dispensary in Paris.

The Dispensary of the *Trente Ans de théâtre* in Paris has benefited by a recent matinée. The Paris papers remind the public that " it is for our poor ones of the theatre removed from it by sickness " that the *Trente Ans de théâtre* exists. We learn that the *Trente Ans de théâtre* have now a dispensary which, under the direction of Professor Pozzi and Dr. Paul Barbarin, has succeeded beyond expectation. During the year 1910 alone the dispensary has given 1,820 free consultations and treatments.

### A Parish Priest as Dispensary Secretary.

An exceedingly good example of the effective value of personal service can be found in the history and present flourishing condition of the Ely dispensary. It was founded in 1862 chiefly through the enthusiasm of Mr. Smith, whose son, the Rev. Kenelm Henry Smith, himself has been its honorary secretary for thirty-one years. During this period Mr. Smith has been closely connected with parish work and local organisations in and about Ely. He was a scholar of St. John's College, Cambridge, was ordained deacon in 1860, and priest in 1864, and from 1863 to 1871 was curate of St. Mary, Ely, and became an assistant minor canon of the cathedral. The dispensary for which he is an enthusiastic worker is well endowed for its size, largely owing to the support that Mr. Smith has been able to gain for it from his friends. Mr. Smith is understood to await with anxiety and determination the effect of the Government's Insurance Bill. "We are worth robbing," he has been known to say, " having a corpus, house property, and investments. But we shall die game." Cambridge men, who remember Ely chiefly by a tea shop in the principal street, still called, perhaps, " Go to sleep," will be delighted to find that the quiet cathedral town contains so active an institution as the dispensary, over the fortunes of which Mr. K. H. Smith presides so energetically.

### Presentation to the Bristol Medical Officer.

The members of the staff of the Health Department at Bristol have presented to Dr. D. S. Davies a piece of plate in commemoration of his twenty-five years' work as medical officer of health for the city. Mr. G. W. Kirley and Mr. S. O. Dimond, both of whom have been associated with Mr. Davies' work for many years, the latter on the port staff, made complimentary speeches, and Dr. Davies, in his reply, said that a quarter of a century, though a long period to look forward to, was a short one to glance back at, and if something of progress had been attained in that time it was not the merit of any one man, but was due to the loyalty, the devotion to a common good end of every member of a staff such as that now gathered around him. Nor must they be satisfied to stand where they were. Progress was never complete, and any present success must lead them on to further endeavour. Furthermore, they must keep in mind the sure foundations laid down for them by their predecessors, by Dr. Budd, whose work on typhoid fever was classical, and by his own father, who enjoyed Dr. Budd's friendship, and who successfully applied his teachings in regard to cholera and the control of disease. The silver coffee service is inscribed : " Presented to D. S. Davies, Esq., M.D., by the entire staff of the Health Department on the twenty-fifth anniversary of his appointment as Medical Officer of Health for the city and port of Bristol." Dr. Davies has just issued his report, which explains Bristol's low death-rate, and emphasises the need for additional hospital beds for isolation cases.

### Legacies for Stockport Hospitals?

It will be interesting to learn whether Major George Fearn, V.D., of Wilmslow, Cheshire, who has left the residue of his property, which appears to amount to over £50,000, for charitable and public purposes in Stockport, has included the Stockport hospitals among the beneficiaries under his will.

### Mr. T. Brittin Archer's Retirement.

At the conclusion of the laying of the foundation-stone of the new building of the Central London Ophthalmic Hospital, the Earl of Stradbroke was asked, on behalf of the surgical and medical staff, and governors, to present to Mr. T. Brittin Archer a silver cup and gold watch as a token of appreciation of his services to the hospital on his retirement from the position of Senior Surgeon. Lord Stradbroke remarked that it was close on forty years ago that Mr. Archer joined the staff of the hospital, and from that time he had ever taken a lively interest in the institution, not only professionally, but in the management and in its financial needs. He had further undertaken the duties of trustee of the Building Fund, and had become lessee of the new premises. His Lordship asked Mr. Brittin Archer's acceptance of the gifts to mark the friendship and esteem in which he was held by all who had the work of the hospital at heart. Mr. T. Brittin Archer was received with applause. "If these gifts mean anything to me," he said, " and they do mean many things—they mean that you recognise that I have done what every Englishman should only be too proud to do—his duty." He referred to the fact that only £8,000 was now required to complete the building scheme, and as their policy was to build a plain but efficient hospital rather than a palatial edifice, he hoped the amount would be forthcoming speedily.

### " The Hospital " v. " The British Medical Journal."

Last Saturday a capital precedent was created in the first cricket match played between The Hospital and the *British Medical Journal*. Batting first, The Hospital side, which included Mr. Palmer (of " Glaxo "), made 148. the *British Medical Journal* then went in and made 47, eight of their side being bowled by Mr. Palmer.

